# Editorial: Plant-Production Platforms for Veterinary Biopharmaceuticals

**DOI:** 10.3389/fpls.2022.858043

**Published:** 2022-02-23

**Authors:** Selene Baschieri, Rima Menassa, Eyal Klement, Marcello Donini

**Affiliations:** ^1^Laboratory of Biotechnology, Italian National Agency for New Technologies, Energy and Sustainable Economic Development (ENEA) Casaccia Research Center, Rome, Italy; ^2^Agriculture and Agri-Food Canada, London Research and Development Centre, London, ON, Canada; ^3^Department of Biology, University of Western Ontario, London, ON, Canada; ^4^Faculty of Agriculture, Food and Environment, Koret School of Veterinary Medicine, The Hebrew University of Jerusalem, Jerusalem, Israel

**Keywords:** veterinary vaccines, veterinary diagnostics, One Health, plant molecular farming, differentiation of infected from vaccinated animals (DIVA)

Public health initiatives such as “One Health” demonstrate the growing awareness that animal and human health are strongly interconnected with each other and with the environment. Veterinary diseases are not only responsible for animal suffering and major economic losses to the livestock industry as well as a threat for endangered species but, when caused by zoonotic pathogens, may also represent a direct danger to human health as demonstrated by the recent COVID-19 pandemic (Fisher and Murray, 2021). Biopharmaceuticals such as vaccines as well as efficient diagnostic assays are the most suitable interventions to prevent and monitor the dissemination of pathogens in both domestic and wild animal populations. However, innovation is needed in this field to increase the efficacy/efficiency of these tools, simplifying delivery and use and reducing manufacturing costs. A wider utilization of novel veterinary vaccines amongst livestock/poultry/fish farmers and producers would have a major impact not only in disease prevention but also in the reduction of the use of chemicals/antibiotics for treatment of many diseases. The number of approved veterinary biotech biopharmaceuticals, mainly vaccines, is still modest when compared to that of analogous products for human use. An important issue hampering the transfer of innovative “medicines” to the veterinary field mainly relates to the generally high manufacturing costs of cutting-edge products that impact on the selling price exceeding in many cases the animal value. This is the reason why for example, currently available veterinary vaccines mainly consist of live attenuated or inactivated viruses. Despite the undeniable advantages of these conventional formulations, some of their features limit their efficacy, including the inability to permit the serological differentiation of infected from vaccinated animals (DIVA) (Aida et al., 2021).

The aim of this Research Topic was to highlight that plants may represent a promising platform for the rapid and low-cost development of innovative recombinant biopharmaceuticals for veterinary use, further reinforcing the numerous examples already present in the literature of the past 20 years describing the use of this platform for the production of a plethora of molecules active against both viral and bacterial pathogens. Plant-based production systems are generally classified according to the adopted plant transformation method (stable or transient) (Rybicki, 2018). Noteworthy, all the research articles collected herein adopt transient transformation technologies that allow to consistently increase yields and reduce overall manufacturing cycle time.

The ability of plants to produce structurally complex proteins with high yields is well-evidenced in the articles by Chin-Fatt et al. and Stander et al. both focused on fighting two zoonotic pathogens representing major public health threats. The first describes a strategy to improve the accumulation of a recombinant antibody designed for passive immunization of cattle herds against Entherohemorragic *Escherichia coli* (EHEC), a pathogen harbored asymptomatically mostly by cattle and a major cause of bloody diarrhea in humans, with dire consequences, especially among children. The latter applies the SpyTag/SpyCatcher conjugation system to produce phage like particles displaying a domain of West Nile Virus (WNV) envelope protein as vaccine antigen against WNV, a mosquito borne virus which might cause encephalitis, both in human and horses. Besides showcasing important technological advancements, these articles highlight the potential of plants as “biofactories” for biopharmaceutical production in supporting the “One Health” concept.

The review by Su et al. illustrates how in the animal husbandry field one of the branches on which Molecular Farming may have the stronger impact is aquaculture. To date, disease prevention in aquatic production is mainly based on the massive use of chemicals and antibiotics rather than relying on the use of vaccines. These chemicals pose serious risks to the environment, human health, and food security. The reason for this trend is mostly attributable to the high costs of the delivery of available fish vaccines, a major problem that could be solved by adopting edible plants as “biofactories.” Edible plants expressing subunit protein vaccines may in fact be directly mixed with feed, offering the potential to stimulate both mucosal and systemic immunity, and reducing production costs while eliminating purification costs of recombinant antigens.

Interesting examples of the efficacy of oral immunization with plant expressed antigens are presented, in which a fractionated extract from plants expressing the VP2 from Infectious Bursal Disease virus (IBDV) (Lucero et al.) or fresh pulverized leaves from plants expressing *Toxoplasma gondii* surface antigen (Sánchez-López et al.) were orally delivered in chickens and in mice, respectively, inducing in both cases the activation of specific and protective immune responses.

Disease management cannot disregard the development of efficient rapid and low-cost diagnostic assays. In this Research Topic, Silva et al. describe the production in plants of the K39 antigen of *Leishmania infantum* fused to hydrophobin in order to simplify and improve purification, resulting in the set-up of a sensitive and specific diagnostic test for dogs.

The potentialities of recombinant subunit vaccines in the veterinary field opens the way also to the development of diagnostic assays allowing DIVA, a strategy not always applicable when inactivated or live attenuated vaccines are used. An example is well-represented here by the work of Bortolami et al. in which the VP3 protein of IBDV expressed in plants allowed to set-up a highly sensitive and specific diagnostic assay able to differentiate infected animals from chickens vaccinated with different types of recombinant vaccines (including VP2-based subviral particles produced in plants).

To summarize, the goal of this Research Topic was to emphasize the possible role of plant “biofactories” as a common playground in which, in line with the One Health Initiative, it is possible to combine a collaborative multidisciplinary effort to face the challenges of developing low-cost, effective, and innovative biopharmaceuticals, as well as novel diagnostic assays for both domestic and wild animals.

## Author Contributions

All authors listed have made a substantial, direct, and intellectual contribution to the work and approved it for publication.

## Conflict of Interest

The authors declare that the research was conducted in the absence of any commercial or financial relationships that could be construed as a potential conflict of interest.

## Publisher's Note

All claims expressed in this article are solely those of the authors and do not necessarily represent those of their affiliated organizations, or those of the publisher, the editors and the reviewers. Any product that may be evaluated in this article, or claim that may be made by its manufacturer, is not guaranteed or endorsed by the publisher.
